# P-208. Does an electronic alert for secondary C. difficile prophylaxis result in vancomycin prophylaxis and reduction in recurrent CDI?

**DOI:** 10.1093/ofid/ofae631.412

**Published:** 2025-01-29

**Authors:** Harjot K Singh, Adam L Gouveia, Barbara Ross, David Kuang, shirley wang, Harold Horowitz, Matthew Simon, Brian Nelson, Heidi M Torres, Steven Kaplan, E Yoko Furuya, David P Calfee

**Affiliations:** Weill Cornell Medicine, new york city, New York; NewYork-Presbyterian, Brooklyn, NY; NewYork-Presbyterian Hospital, New York, NY; NEW YORK PRESBYTERIAN, HOBOKEN, New Jersey; NYP, new york, New York; New York Presbyterian-Brooklyn Methodist Hospital, Brooklyn, New York; Weill Cornell Medicine, new york city, New York; NewYork-Presbyterian Hospital, New York, NY; Weill Cornell Medicine, new york city, New York; NewYork-Presbyterian, Brooklyn, NY; Columbia University Irving Medical Center and NewYork-Presbyterian Hospital, New York, NY; Weill Cornell Medicine, new york city, New York

## Abstract

**Background:**

Secondary vancomycin prophylaxis (VP) is one strategy to reduce recurrent *C. difficile* infection (CDI). Clinical decision support (CDS) was implemented in the electronic health record (Epic®) in 2020 to remind providers about eligibility for VP at time of prescription of broad-spectrum antibiotics in patients with a history of CDI. The effectiveness of this strategy was assessed.

CDIFF BPA

**Figure:**
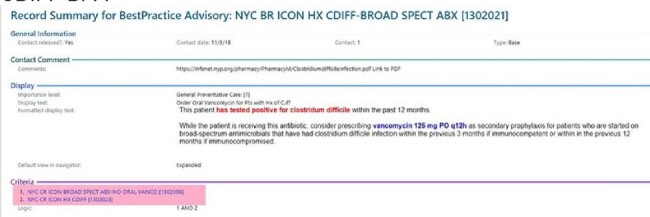

CDIFF Secondary Prophylaxis Best Practice Alert

**Methods:**

Retrospective review of an interruptive best practice alert (BPA) for VP at 8 hospitals from 2021-2022. Patients were classified via manual chart review as immunocompetent (immune) or immunocompromised, and were eligible for 3 months and 12 months respectively after an episode of CDI. Demographic characteristics, immune status, receipt of VP, and outcomes (30-day recurrence CDI, death during hospitalization or within 30 days of discharge) were collected. Only the first BPA per patient was included in the demographic and death analysis. Only one alert per hospitalization was included in the VP use and recurrence analysis. Patients with CDI within 10 days prior to the BPA and on the same day as BPA alert were excluded. Descriptive statistics were used to analyze differences.

**Results:**

Among 616 unique hospitalized patients for whom the BPA was triggered, median age was 64 years; 40% were immune. Approximately 50% were women, with 24% African American and 24% Hispanic. After 1562 BPAs, 13% of immunocompromised and 10% of immune patients were prescribed VP within 24 hours. Median days from previous CDI to BPA was 97 days for immunocompromised and 45 days for immune patients. For immunocompromised patients, frequency of CDI recurrence (11% versus 6%) and frequency of death (14% versus 18%) were not statistically different in the group that got VP and the group that didn’t. Similarly for immune patients, frequency of recurrent CDI (7% versus 8%) or death (16% versus 13%) were not statistically different in the group that got VP and the group that didn’t.

**Conclusion:**

VP was infrequently initiated despite an interruptive BPA that identified patients with recent history of CDI at the time of prescription of broad-spectrum antibiotics. In this limited sample size, patients had similar outcomes regardless of VP. Further research is needed to understand the barriers for the low uptake, including CDS design and efficacy of VP.

**Disclosures:**

**All Authors**: No reported disclosures

